# Albumin fusion with granulocyte-macrophage colony-stimulating factor acts as an immunotherapy against chronic tuberculosis

**DOI:** 10.1038/s41423-020-0439-2

**Published:** 2020-05-07

**Authors:** Yu-Min Chuang, Liangmei He, Michael L. Pinn, Ya-Chea Tsai, Max A. Cheng, Emily Farmer, Petros C. Karakousis, Chien-Fu Hung

**Affiliations:** 1 Department of Pathology, Johns Hopkins University School of Medicine, Baltimore, MD USA; 2 Department of Medicine, Johns Hopkins University School of Medicine, Baltimore, MD USA; 3 Present Address: Section of Infectious Diseases, Department of Internal Medicine, Yale University School of Medicine, New Haven, CT USA

**Keywords:** Tuberculosis, albumin, GM-CSF, therapeutic vaccine, fusion protein, Immunology, Immunotherapy

## Abstract

A long duration of treatment and emerging drug resistance pose significant challenges for global tuberculosis (TB) eradication efforts. Therefore, there is an urgent need to develop novel strategies to shorten TB treatment regimens and to treat drug-resistant TB. Using an albumin-fusion strategy, we created a novel albumin-fused granulocyte-macrophage colony-stimulating factor (albGM-CSF) molecule that harnesses albumin’s long half-life and targeting abilities to enhance the biostability of GM-CSF and direct it to the lymph nodes, where the effects of GM-CSF can increase dendritic cell populations crucial for eliciting a potent immune response. In this study, we demonstrate that albGM-CSF serves as a novel immunotherapy for chronic *Mycobacterium tuberculosis* (*Mtb*) infections by enhancing GM-CSF biostability in serum. Specifically, albumin is very safe, stable, and has a long half-life, thereby enhancing the biostability of GM-CSF. In the lungs and draining lymph nodes, albGM-CSF is able to increase the numbers of dendritic cells, which are crucial for the activation of naive T cells and for eliciting potent immune responses. Subcutaneous administration of albGM-CSF alone reduced the mean lung bacillary burden in mice with chronic tuberculosis infection. While GM-CSF administration was associated with IL-1β release from *Mtb*-infected dendritic cells and macrophages, higher IL-1β levels were observed in albGM-CSF-treated mice with chronic tuberculosis infection than in mice receiving GM-CSF. Albumin fusion with GM-CSF represents a promising strategy for the control of chronic lung tuberculosis infections and serves as a novel therapeutic vaccination platform for other infectious diseases and malignancies.

## Introduction

Tuberculosis (TB) is currently the most common cause of death by a single infectious agent worldwide.^1^ Efforts have been made to implement a 6-month “short-course” combination regimen for the treatment of drug-susceptible TB. Although this regimen has been shown to be efficacious, it requires proper provision and direct supervision, which can be taxing for health care systems, especially in TB-endemic regions. Inadequate treatment of TB leads to excess morbidity and mortality, continued transmission, and emergence of drug resistance.^2^ Therefore, novel strategies are needed to shorten the duration of curative TB treatment. In addition to novel antimicrobial agents, host-directed therapies represent attractive strategies to combat disease due to drug-susceptible and drug-resistant *Mycobacterium tuberculosis* (*Mtb*).^3^ In particular, host-directed therapies may reverse TB-related lung inflammation and/or augment innate and adaptive immune responses to accelerate mycobacterial clearance during anti-TB treatment.^4^

Effective immunity against TB depends on antigen presentation by MHC class I or class II molecules, which occurs in the draining lymph nodes (dLNs) at the site of infection.^5^ Dendritic cells (DCs) are key antigen-presenting cells (APCs) that activate naive T cells by upregulating chemokine receptors and costimulatory molecules.^5,6^ Mature DCs are characterized by higher expression of surface MHC class II molecules and the integrin-αX chain, as well as CD11c and other costimulatory molecules.^7^ Adoptive transfer of antigen-pulsed DCs has been shown to significantly improve vaccination efficacy relative to control treatment, indicating that adequate antigen presentation in the lungs is one of the key factors for controlling *Mtb* infection.^8^

Granulocyte-macrophage colony-stimulating factor (GM-CSF) is a hematopoietic growth factor critical for DC generation, proliferation, and maturation.^9–11^ Other myeloid lineage cells, including monocytes, macrophages, neutrophils, and eosinophils, are also activated by GM-CSF.^9^ Coadministration of GM-CSF during vaccination increases antigen-specific IFNγ-secreting T cells and enhances protection against various infectious agents.^12–18^ Conversely, deficiency of GM-CSF is associated with reduced T-cell responses after vaccination.^19^ Mice vaccinated with a bacillus Calmette–Guérin vaccine including cells expressing murine GM-CSF were found to have enhanced DC maturation in dLNs and increased protection against disseminated TB.^16^ In addition to its promotion of the maturation of DCs, GM-CSF is one of the key cytokines that promotes the differentiation of M1 macrophages, which are key effectors in controlling intracellular pathogens through the release of proinflammatory cytokines.^20–22^ GM-CSF enhances the ability of human macrophages to inhibit *Mtb* growth ex vivo.^23^ GM-CSF^−/−^ mice are highly susceptible to *Mtb* infection, and anti-GM-CSF autoantibodies increase the risk of cryptococcal meningitis and pulmonary TB in patients.^10,24–26^ GM-CSF secreted by T cells has been shown to offer protection against *Mtb* infection in murine models.^25^ A greater proportion of GM-CSF^+^ multifunctional CD4^+^ T cells are present in latently infected individuals than in those with active TB.^27^ Furthermore, GM-CSF secretion was significantly reduced when CD3^+^ T cells were cocultured with myeloid-derived suppressor cells in patients with active TB.^28^ These observations indicate that GM-CSF plays an important role in innate immunity and initiating adaptive immunity, indicating the potential utility of this cytokine in anti-TB immunotherapy. Indeed, GM-CSF enhances the bactericidal activity of anti-TB drugs in both mouse models and in humans.^29–31^ However, the observed synergy of GM-CSF is relatively limited due to its side effects, short half-life of ~7 h, and reduced penetration into the lungs.^32^ Therefore, an alternative strategy is required to improve the bioavailability of GM-CSF in the lungs, which are the primary site of TB.

A fusion strategy using the fragment crystallizable region (Fc region) has been used to improve the biostability and half-life of proteins, as well as for mucosal targeting.^33–35^ However, one limitation of this approach is the potential development of autoantibodies directed against the Fc region.^33,34^ Similar to immunoglobulin, albumin has an extended serum half-life of 3 weeks due to its size and its ability to undergo neonatal Fc receptor (FcRn)-mediated recycling, thus preventing intracellular degradation.^33^ Since albumin is very safe and stable and has a very long half-life, it has been frequently used for drug delivery. Currently, there are six albumin-based drugs that are commercially available, with many more being tested in clinical trials.^36^ Labeled human albumin has been used for LN identification and imaging, suggesting that albumin can traffic to LNs.^37^ We reasoned that these favorable properties of albumin may be exploited by fusing this protein to GM-CSF to enhance the serum levels of GM-CSF and augment its effect in the lungs and LNs, thus achieving organ-targeting vaccination. Specifically, we determined whether murine albumin conjugation was able to increase the effects of GM-CSF in mice. In our proof-of-concept studies, we show that this albumin-fusion strategy (albGM-CSF) enhances the serum levels of GM-CSF, leading to increased DC populations, cytokine secretion, and CD4^+^ T-cell activation, thus improving the control of chronic TB in mice.

## Materials and methods

### DNA constructs and protein expression

To generate albumin-fused GM-CSF (albGM-CSF), mouse albumin was first amplified with PCR using the cDNA template of mouse albumin (AAH49971, transOMIC Technologies, Huntsville, AL, USA) and a set of primers, 5′-AAATCTAGAGCCACCATGAAGTGGGTAACCTTT-3′ and 5′-TTTGAATTCGGCTAAGGCGTCTTTGCATC-3′. The amplified product was then cloned into the XbaI/EcoRI sites of a pcDNA3 vector (Invitrogen Corp., Carlsbad, CA, USA). Next, for the generation of pcDNA3-AlbGM-CSF, mouse GM-CSF was first amplified via PCR with a cDNA template of the mouse GM-CSF (NM_009969.4) gene synthesized from Genscript (Piscataway, NJ, USA) and the following primers: 5′-TTTGAATTCGCACCCACCCGCTCACCCAT-3′ and 5′-AAACTTAAGTCATTTTTGGACTGGTTTTTTG-3′. The amplified product was then cloned into the EcoRI/Afl II sites of pcDNA3-Alb. For the generation of pcDNA3-albGLuc, Gaussia luciferase (GLuc) was first amplified via PCR with a cDNA template of phGLuc (gifted from Dr John Schiller, NIH) and the primers 5′-AAAGAATTCATGGGAGTCAAAGTTCTGTTTG-3′ and 5′-TTTAAGCTTTTAGTCACCACCGGCCCCCTTG-3′. The amplified product was then cloned into the EcoRI/HindIII sites of pcDNA3-Alb. For the generation of pET28a-GLuc, GLuc was first amplified via PCR with a cDNA template of phGLuc and the following primers: 5′-AAAGAATTCGAGGCCAAGCCCACCGAGAAC-3′ and 5′-TTTCTCGAGGTCACCACCGGCCCCCTTGA-3′. The amplified product was then cloned into the EcoRI/XhoI sites of the PET28a vector (Novagen Inc., Madison, WI, USA). All plasmid constructs were confirmed by DNA sequencing. The AlbGM-CSF and albumin-GLuc (albGLuc) proteins were expressed using the Expi293F Expression System Kit (Thermo Fisher Scientific, Waltham, MA, USA) according to the manufacturer’s instructions. Expi293F cells were transfected with albGM-CSF and alb-GLuc, and the transfection efficiency was determined by the expression levels of the target protein. Proteins were purified by a HiTrap albumin column (GE Healthcare Life Sciences, Marlborough, MA, USA). GLuc was expressed in *E. coli* BL21 (Rosetta cells; Novagen) and purified by Ni^+^ affinity chromatography (Ni-NTA agarose, Qiagen Sciences, Germantown, MD, USA) according to the manufacturer’s protocol). Mouse GM-CSF was purchased from Genscript.

### In vivo GLuc activities

Eight-to-ten-week-old female C57BL/6J (*n* = 3–4) (NCI, Frederick, MD, USA) or eight-to-ten-week-old FcRn-knock-out (KO) mice (*n* = 3–4) (B6.129X1-*Fcgrt*^tm1Dcr^/DcrJ, Jackson Laboratory, Bar Harbor, ME, USA) received retro-orbital injections with either Gaussia luciferase (GLuc) (20 μg) or albGLuc (2.8 μg) in 20 μL of phosphate-buffered saline (PBS) following anesthesia by ketamine/xylazine intraperitoneal injection. Seventy-two hours after injection, mice were euthanized, and the serum, inguinal LNs, and lungs were removed and disrupted by bead-beating. GLuc activity was measured with coelenterazine-H (Regis) by a GloMax Luminometer (Promega, Madison, WI, USA). The total luminescence was normalized to tissue weight.

### Mice

The mice were housed in the Oncology Center Animal Facility at the Johns Hopkins Medical Institutes (Baltimore, MD, USA). All animal procedures were performed according to the approved protocols and in accordance with the recommendations for the proper use and care of laboratory animals. To ensure that animal discomfort, distress, pain, and injury were kept to a minimum, a maximum of five mice were housed in the same cage. All animals were maintained and all experiments were performed according to the protocols approved by the Institutional Animal Care and Use Committee at the Johns Hopkins University School of Medicine.

### Lung injury model

Female C57BL/6J mice (6–8 weeks old; *n* = 3–4) received 20 μg of lipopolysaccharide (LPS) from *E. coli* O26:B6 (Sigma-Aldrich, St. Louis, MO, USA) via the intranasal route. The control group received PBS. One day later, both groups of mice received either GLuc (20 μg) or albGLuc (2.8 µg) by intranasal injection. To measure luciferase activity, sera from each group were collected the following day. Luciferase expression was taken to indicate transcytosis activity in the airway, as previously described.^38^

### Mycobacteria and growth conditions

Wild-type *Mtb* H37Rv was grown in Middlebrook 7H9 broth (Difco, Sparks, MD, USA) supplemented with 10% oleic acid-albumin-dextrose-catalase (Difco), 0.1% glycerol, and 0.05% Tween-80 at 37 °C in a roller bottle.^39^

### Bone marrow-derived macrophage (BMDM) and bone marrow-derived DC (BMDC) cell assays

Bone marrow was harvested from C57BL/6J mice, as previously described.^40^ To compare the efficacy of GM-CSF and albGM-CSF, bone marrow cells were incubated with either GM-CSF (0.625 µM) or albGM-CSF (0.625 µM) at 37 °C in 5% CO_2_ and RPMI media with 10% fetal bovine serum (FBS) for 7 days (Sigma-Aldrich). Antigen-containing supplemented media were replenished on day 3. The cells were collected to analyze the percentage of CD11c^+^ cells and CD11c^+^MHCII^+^ cells. For *Mtb* infection studies, bone marrow cells were incubated with GM-CSF (10 ng/mL, Genscript) to obtain BMDCs or with macrophage colony-stimulating factor (10 ng/mL, Genscript) to obtain BMDMs. A total of 2 × 10^5^ BMDMs or 5 × 10^5^ BMDCs were plated in a 24-well plate 1 day prior to infection. H37Rv was used to infect BMDCs at an MOI of 1:2.5 (1.25 × 10^6^ bacteria) for 1 day or BMDMs (5 × 10^5^ bacteria) for 2 days in 1 mL of complete RPMI medium (Gibco Laboratories, Gaithersburg, MD, USA) with 10% FBS (Sigma-Aldrich) and 0.625 µM GM-CSF or albGM-CSF.

### Enzyme-linked immunosorbent assays (ELISAs) for GM-CSF or IL-1β

The levels of GM-CSF in sera and IL-1β in cell culture media were determined by ELISAs with mouse GM-CSF or IL-1β DuoSet ELISA kits from R&D Systems (Minneapolis, MN, USA). The IL-1β levels of lung lysates were normalized to the lysate protein concentration using a Qubit protein assay kit (Thermo Fisher Scientific).

### Intracellular cytokine stains and flow cytometry analyses

At predetermined time points, the mice were euthanized, and cells from LNs and spleens were collected, as previously described.^41,42^ To determine ESAT6 antigen-specific CD4^+^ or TB10 antigen-specific CD8^+^ T-cell responses, splenocytes were incubated with ESAT6 ((MTEQQW NFAGIEAAA) or TB10 (IMYNYPAM) peptides (Genscript) and GolgiPlug (BD Biosciences, San Jose, CA, USA) overnight.^43^ After incubation, the cells were washed once with FACScan buffer and then stained with a PE-conjugated monoclonal rat anti-mouse CD4 antibody (BD Biosciences) and/or an APC-conjugated monoclonal rat anti-mouse CD8 antibody (eBioscience, Inc., San Diego, CA, USA). Cells were permeabilized using the Cytofix/Cytoperm kit (BD Biosciences). Intracellular IFN-γ was stained using a FITC-conjugated rat anti-mouse IFN-γ antibody. Flow cytometry was performed on a FACSCalibur instrument, and the results were analyzed with FlowJo software (Supplementary Fig. 1). To collect pneumocytes, the lungs were perfused with 1 mL of normal saline by direct injection into the right ventricle of the heart at necropsy. A section of the lung was used for cytometry analysis, and the tissue samples were incubated at 37 °C for 1 h with intermittent agitation in RPMI medium (Gibco Laboratories) containing collagenase D (1 mg/mL, Sigma-Aldrich), DNAase (0.25 mg/mL, Sigma-Aldrich), and hyaluronidase type V (1 mg/mL, Sigma-Aldrich). The cells were then filtered through a 70-μm nylon filter mesh to remove undigested tissue fragments and washed with complete RPMI medium. To identify surface markers of DCs from lungs, spleens, or LNs, PE-conjugated anti-mouse MHCII, FITC-conjugated anti-mouse CD11c, APC-conjugated anti-mouse CD103, APC-conjugated anti-mouse CD11b, and APC-conjugated anti-mouse DEC205 (eBioscience, Inc.) antibodies were used to stain cells from each of these tissues (Supplementary Figs. 2 and 3). After washing with FACScan buffer, the cells were counted with a FACSCalibur and analyzed using FlowJo software (Supplementary Figs. 2 and 3).

### Aerosol infection of mice with *Mtb*

Female 6–8-week-old C57BL/6J mice were aerosol-infected with ~100 bacilli of wild-type *Mtb* H37Rv. After 1 month of infection, groups of mice received human-equivalent doses of isoniazid (10 mg/kg) by esophageal gavage once daily (5 days/week). In the experimental groups, mice received 0.375 μM GM-CSF or albGM-CSF in 100 µL of PBS by retro-orbital or subcutaneous injection at predetermined time points. Mice were euthanized on days 30, 60, and 90 after aerosol challenge, lungs were homogenized, and the cells were plated for colony-forming unit assays to evaluate the potential synergistic effect of each therapeutic vaccine.^44^

### Statistical analyses

Mean differences between groups were compared using one-way analysis of variance with Tukey–Kramer post hoc analyses (MedCalc software, Ostend, Belgium). If the data did not pass through the normality test by the D’Agostino–Pearson test, the differences of groups were compared by the Kruskal–Wallis test with post hoc analyses. Data from at least three biological replicates were used to calculate means and standard errors of the mean (SEMs) for graphing purposes. To compare differences between experimental and control groups, statistical analyses employed the Mann–Whitney test for sample sizes less than 4 or an unpaired Student’s *t* test for sample sizes >4, and a *p* value of < 0.05 was considered statistically significant.

## Results

### Albumin fusion increases protein biostability and targets the protein to the lungs and LNs

**Fig. 1:**
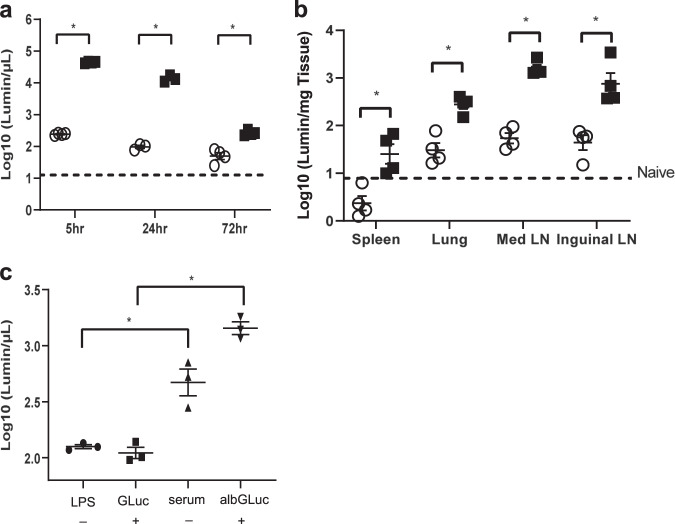
Albumin fusion increases the biostability and targeting of proteins to the lungs and LNs. Either 20 µg of Gaussia luciferase (GLuc, white bar) or 2.8 µg of albumin-fused Gaussia luciferase (albGLuc, black bar) were intravenously injected into C57BL/6J mice. Both reagents had similar luciferase activities before injection. **a** At different time points after injection, serum luciferase activities were determined in both groups (*n* = 4). **b** Seventy-two hours after either GLuc or albGLuc injection, the mice were euthanized, and their spleens, lungs, and LNs were harvested. Luciferase activities were measured from the tissue lysates and then normalized by individual tissue weights (*n* = 4). **c** Twenty micrograms of either LPS or PBS was administered to the lungs by intranasal injection. Twenty-four hours later, either GLuc or albGLuc was given via the intranasal route for another 24 h. Sera were then collected to determine luciferase activities (*n* = 3). All data are represented as the mean ± SEM, *p < 0.05

Previous studies have reported that fusing albumin to various proteins can be used to image LNs.^37^ In the current study, we used murine albumin-fused GLuc (albGLuc) to determine the tissue distribution and serum levels of GLuc. Separate groups of female C57BL/6J mice received retro-orbital injections with either GLuc or albGLuc. In the experiments to analyze the enzymatic activity of the protein in different tissues, both albGLuc (0.028 µg/µL) and GLuc (0.2 µg/µL) had similar luminescence activities prior to injection (1.514 × 10^10^ vs. 3.981 × 10^10^ luminescence/mL, respectively). After injection of albGLuc or GLuc, the serum luciferase activity of the albGLuc group was 100-fold higher than that of the GLuc group 24 h after injection, and this significant difference in serum luciferase activity was maintained for at least 72 h after injection (Fig. 1a). The luciferase activity was at least fivefold higher in the inguinal and mediastinal LNs of mice injected with albGLuc than in mice injected with GLuc alone after 72 h of injection (Fig. 1b). The luciferase activity of the lungs was significantly (tenfold) higher in the albGLuc group than in the GLuc group after 72 h of injection. Therefore, the albumin-fusion strategy appears to significantly increase the levels of target protein in the serum, with enhanced delivery to the LNs and lungs.

FcRn is required for the recycling and exocytosis of albumin-fused proteins.^33,45^ However, there is limited information about the activity of FcRn in chronic pulmonary TB. To understand the role of FcRn in transcytosis during pulmonary inflammation, we delivered albGLuc to mice by intranasal injection under inflammatory conditions and subsequently measured serum luciferase activities through the airway epithelium as an index of protein transcytosis. In mice with lung injuries induced by the intranasal injection of LPS 24 h before albGLuc injection, serum GLuc activity was found to be significantly higher in the group receiving intravenous injection of albGLuc than in the group receiving GLuc alone (Fig. 1c). Based on these observations, we conclude that the albumin-fusion strategy offers a promising approach for delivering target proteins to acutely and chronically inflamed mouse lungs.

### albGM-CSF increases the number of DCs in the dLNs and lungs

**Fig. 2:**
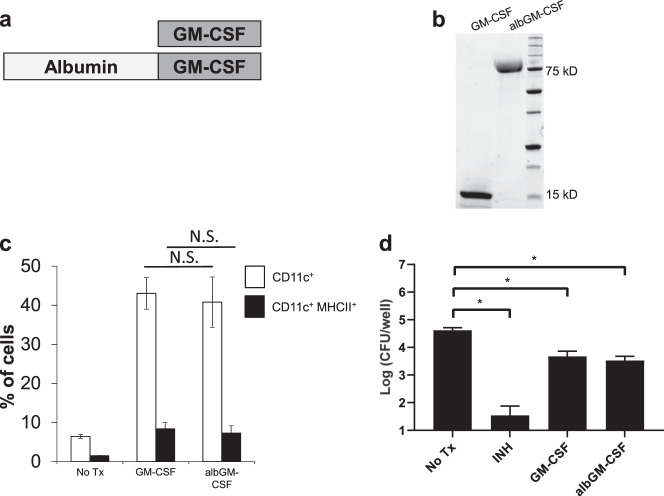
Both GM-CSF and albGM-CSF kill intracellular *Mtb* in infected macrophages. GM-CSF was fused with albumin to increase its half-life and tissue penetration. **a** Schematic of the albumin-GM-CSF fusion protein. **b** Albumin-GM-CSF (albGM-CSF) protein was purified by SDS-PAGE. **c** To test the biological properties of albGM-CSF, BM cells were collected from C57B/6J mice and stimulated with 0.625 µM GM-CSF or albGM-CSF for 7 days. The percentage of DCs was determined by flow cytometry using the markers CD11c and MHCII. **d** GM-CSF and albGM-CSF demonstrated significant *Mtb* killing in infected macrophages. All data are represented as the mean ± SEM, *n* = 3, *p < 0.05

**Fig. 3:**
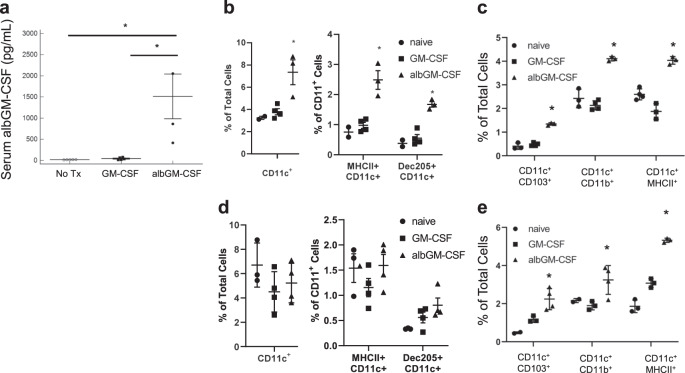
Albumin fusion increases serum levels of GM-CSF and albGM-CSF and increases the number of DCs in the dLNs and lungs in vivo. **a** C57BL/6J mice were subcutaneously injected with either GM-CSF or albGM-CSF. One day after injection, the serum level of GM-CSF was determined by ELISA (*n* = 5 for the naive and GM-CSF groups, *n* = 4 for the albGM-CSF group). GM-CSF or albGM-CSF was subcutaneously injected into C57BL/6J mice. Five days later, the mice were euthanized, and DCs were quantified by flow cytometry in the **b** dLNs and **c** lungs. To determine the role of FcRn in the metabolism of albGM-CSF, either GM-CSF or albGM-CSF was subcutaneously injected into FcRn-KO mice. Five days later, the mice were euthanized, and DCs were quantified by flow cytometry in the **d** dLNs and **e** lungs (*n* = 4 for the GM-CSF group, *n* = 3–4 for the albGM-CSF group). All data are represented by the mean ± SEM, *p < 0.05

Studies have shown that GM-CSF enhances the intracellular killing of bacteria in vitro and that it is a key cytokine secreted by invariant natural killer (iNK) T cells to control intracellular *Mtb* growth.^46,47^ To test whether a murine albumin-fusion strategy can enhance *Mtb* control during chronic infection, we generated albGM-CSF, which was expressed in Expi293F cells and purified by an albumin-binding column (Fig. 2a, b). We used GM-CSF and albGM-CSF to stimulate mouse bone marrow-derived cells ex vivo for 7 days. Both GM-CSF and albGM-CSF induced the differentiation of bone marrow cells into mature DCs without significant differences between the two groups (Fig. 2c). It has been shown that GM-CSF can directly inhibit *Mtb* growth in macrophages.^47^ To test whether albGM-CSF has the same effects, we incubated *Mtb*-infected macrophages with either GM-CSF or albGM-CSF (Fig. 2d). Both albGM-CSF and GM-CSF significantly decreased *Mtb* growth in macrophages. To determine whether albumin fusion enhances the stability of GM-CSF in serum, mice received either GM-CSF or albGM-CSF by subcutaneous injection. One day after injection, the level of serum GM-CSF was significantly higher in mice receiving albGM-CSF than in those receiving GM-CSF (Fig. 3a). To investigate whether albGM-CSF could also induce DC maturation in vivo, either albGM-CSF or GM-CSF was subcutaneously injected into C57BL/6J mice. The number of DCs detected in the dLNs of mice 5 days after injection was significantly increased in mice that received albGM-CSF relative to those that received GM-CSF or no treatment (Fig. 3b). Similarly, the lungs of mice injected with albGM-CSF contained significantly higher numbers of DCs and mature DCs than those of control mice (Fig. 3c). Therefore, albumin fusion of GM-CSF increases the number of DCs in the LNs and lungs of mice, providing a novel strategy to enhance immunity against *Mtb* infection.

As previously mentioned, FcRn is required for immunoglobulin G (IgG) recycling and transport to LNs and prolongs the half-life of both albumin and IgG.^48–50^ To determine whether FcRn is required for albGM-CSF to mediate the enhancement of DC maturation in the LNs or lungs, FcRn-KO mice and C57BL/6J mice were subcutaneously injected with either albGM-CSF or GM-CSF. Four days after injection, the numbers of DCs observed in the dLNs of C57BL/6J mice receiving albGM-CSF were significantly increased compared with those observed in mice receiving GM-CSF alone (Fig. 3b), but there was no such difference between albGM-CSF or GM-CSF treatments in the FcRn-KO mice (Fig. 3d). Interestingly, the number of DCs in the lungs of FcRn-KO mice was still significantly higher than that in the albGM-CSF-treated group, indicating an alternative mechanism mediating the recruitment of DCs to the lungs by albGM-CSF. Therefore, albGM-CSF-mediated DC recruitment to dLNs is dependent on the presence of FcRn (Fig. 3e).

### Albumin fusion enhances GM-CSF-mediated control of chronic *Mtb* infection in mice

**Fig. 4:**
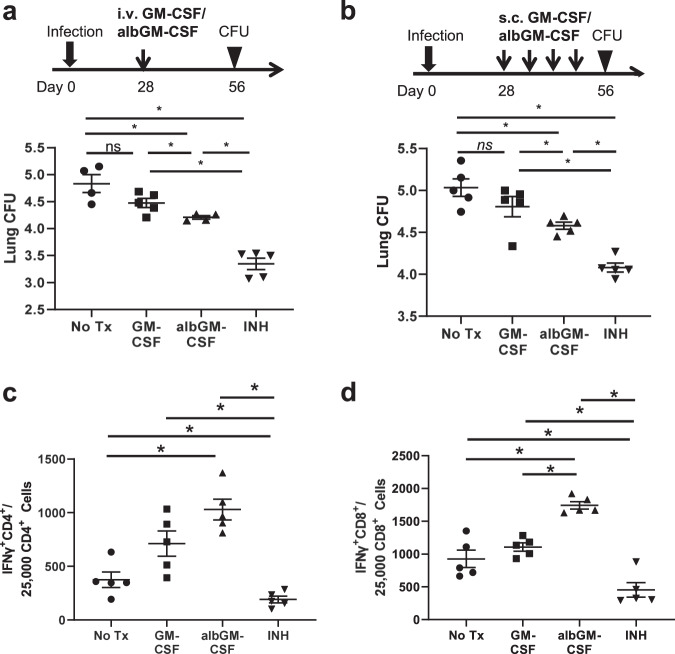
AlbGM-CSF administration enhances lung bacillary control during chronic TB. **a** After 28 days of low-dose aerosol *Mtb* exposure, 0.375 µmol of albGM-CSF or GM-CSF was given by retro-orbital injection once. Four weeks after injection, the lung bacillary colony-forming units (CFUs) were determined (*n* = 4 for the untreated and albGM-CSF groups, *n* = 5 for the GM-CSF and INH groups). **b** In a separate experiment, either albGM-CSF or GM-CSF was given by subcutaneous injection to mice once weekly for 4 weeks after 28 days of low-dose aerosol *Mtb* exposure. Four weeks after the first injection, the lung CFUs were determined. All data are represented as the mean log_10_ CFU ± SEM, *n* = 5, *p < 0.05; NS not significant. After four subcutaneous injections, the lung cells were collected from each group and then stimulated with ESAT6 (**c**) or TB10.4 (**d**) peptides overnight. Antigen-specific CD4^+^ (**c**) or CD8^+^ (**d**) T cells were analyzed by intracellular IFNγ release assay and flow cytometry. All data are represented as the mean, *n* = 4, *p < 0.05

To determine whether albumin fusion increases the immunity induced by GM-CSF during chronic TB in mice, 4 weeks after aerosol infection with *Mtb*, female C57BL/6J mice were intravenously injected once with albGM-CSF or GM-CSF. One group of mice was treated daily (5 days/week) with isoniazid by esophageal gavage.^42^ The control group received no treatment. Four weeks after treatment initiation, the mice were euthanized to determine the lung bacterial burden. The lungs of the albGM-CSF-treated group had a significantly lower mean bacterial burden than those of the untreated and GM-CSF-treated groups (*p* < 0.01; Fig. 4a). Although GM-CSF alone appeared to reduce the mean lung bacterial burden relative to no treatment, consistent with prior reports,^46,47^ this difference was not statistically significant. To determine whether the anti-TB activity of albGM-CSF was dependent on the intravenous route of administration, female C57BL/6J mice were first infected with *Mtb* by an aerosol route, and 4 weeks later, they were subcutaneously injected with either albGM-CSF or GM-CSF once every week for 4 weeks (Fig. 4b). As in the case of intravenous injection, the mean lung bacillary counts were significantly lower in mice receiving subcutaneous injection of albGM-CSF than in those receiving no treatment or GM-CSF. GM-CSF treatment did not significantly change the bacillary burden in the lungs compared with no treatment. There was no significant difference in the gross pathology and lung weights between the albGM-CSF-treated, GM-CSF-treated, and untreated groups (data not shown). Therefore, albGM-CSF, delivered via either the intravenous or subcutaneous route, offers superior therapeutic efficacy against chronic TB relative to GM-CSF or no treatment (Fig. 4a, b).

Given the recognized role of hematopoietic growth factors in adaptive immune responses,^12,16^ we next harvested intrapulmonary lymphocytes from chronically infected mice subcutaneously injected with albGM-CSF or GM-CSF and stimulated them with peptides from the immunodominant antigens ESAT6 and TB10.4 to measure antigen-specific CD4^+^ and CD8^+^ T cells by intracellular cytokine staining. The lungs of mice receiving albGM-CSF, despite harboring a lower bacillary burden, contained significantly more ESAT6-specific CD4^+^ and TB10.4-specific CD8^+^ T cells than those of the untreated group (Fig. 4c, d). Compared with those of the GM-CSF-treated group, the lungs of the albGM-CSF-treated group showed significantly higher numbers of TB10.4-specific CD8^+^ T cells. At the same time, the isoniazid-treated group, which had a significantly lower mean lung bacillary burden, had significantly fewer ESAT6-specific CD4^+^ and TB10.4-specific CD8^+^ T cells than the no-treatment control group. This finding is further supported by previous studies.^51,52^ Taken together, our data highlight that subcutaneous albGM-CSF plays a potential immunotherapeutic role through induction of antigen-specific T cells in the lungs.

### GM-CSF increases the release of the proinflammatory cytokine IL-1β ex vivo and in vivo

**Fig. 5:**
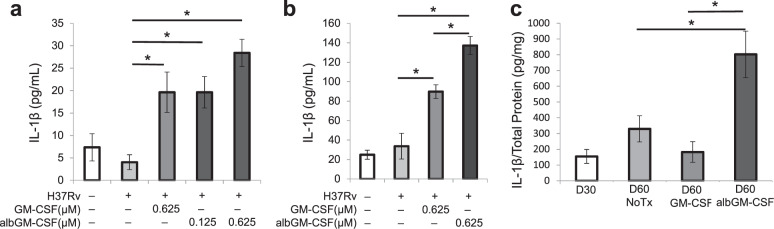
AlbGM-CSF increases the release of IL-1β from Mtb-infected DCs and macrophages. **a** BMDMs (2 × 10^5^/well) were infected with *Mtb* H37Rv (MOI = 1:2.5) for 1 day. Either GM-CSF or albGM-CSF was added to each well as indicated after infection. The supernatant was collected, and IL-1β was measured by ELISA. **b** BMDCs (5 × 10^5^) were infected with *Mtb* H37Rv (MOI = 1:2.5) for 2 days. Either GM-CSF or albGM-CSF was added to each well as indicated after infection. The supernatant was collected, and IL-1β expression was measured by ELISA. All data are represented by the mean ± SEM, *n* = 3, *p < 0.05. **c** After 28 days of low-dose aerosol *Mtb* exposure, albGM-CSF or GM-CSF was given by subcutaneous injection once weekly for 4 weeks. Four weeks later, the lungs were harvested and homogenized. IL-1β release in lung lysates was measured by ELISA and then normalized to the total protein concentration of the lysate. All data are represented as the mean ± SEM, *n* = 5, *p < 0.05

Macrophages and DCs express the GM-CSF receptor, whereas T cells do not,^53^ and both can be infected by *Mtb*.^54–57^ GM-CSF enhances LPS-induced IL-1β secretion from DCs and macrophages by promoting NF-κB signaling.^58,59^ IL-1β plays a key role in the control of intracellular *Mtb* infection.^47^ To determine whether GM-CSF can enhance IL-1β secretion during *Mtb* infection, murine BMDMs were infected with *Mtb* H37Rv ex vivo and exposed to 0.625 µM albGM-CSF or GM-CSF. Both GM-CSF and albGM-CSF exposure enhanced IL-1β secretion from BMDMs after overnight infection (Fig. 5a). Next, murine BMDCs were infected with *Mtb* and exposed to 0.625 µM albGM-CSF or GM-CSF. Both groups showed higher IL-1β secretion (Fig. 5b). Finally, we sought to determine whether albGM-CSF injection in vivo could enhance IL-1β secretion in *Mtb*-infected mouse lungs. As expected, mice chronically infected with *Mtb* receiving albGM-CSF had significantly higher IL-1β levels in the lungs after normalization with total protein lysate than those receiving GM-CSF (Fig. 5c). Taken together, our findings demonstrate that GM-CSF enhances IL-1β secretion from macrophages and DCs and that administration of albGM-CSF to *Mtb*-infected mice induces more IL-1β in the lungs.

## Discussion

Host-directed therapy for TB has generated considerable enthusiasm as a potential approach to improve or reverse TB-induced lung damage, as well as shorten the treatment course for drug-susceptible and drug-resistant disease. However, several important challenges remain before such therapies can be used in the clinical setting, including the need to ensure their bioavailability at the primary site of infection, the lungs.^3^ Prior work has shown that iNK T cells control intracellular *Mtb* infection by secreting the antimicrobial cytokine GM-CSF.^47^ In the current study, we used an albumin-fusion strategy to enhance delivery of GM-CSF to the lungs and dLNs. We found that albGM-CSF increased DCs in the dLNs and yielded an antitubercular effect ex vivo and in mouse lungs.

FcRn, a cell membrane-bound receptor, is expressed in endothelial cells^60^ and professional APCs^61^ in the intestines, lungs, and kidneys.^48^ Endocytosis and transcytosis through FcRn are believed to play a key role in IgG recycling and transport to LNs.^48^ Albumin binds to FcRn, which may increase its half-life.^49,50^ During TB, FcRn is required for IgG homeostasis and CD103^+^ DCs in the lungs.^62^ FcRn-targeting vaccines have shown promising immunological responses, including antibody- and antigen-specific CD4^+^ and CD8^+^ T-cell responses.^35^ It has been shown that the half-life of GM-CSF is less than 24 h in humans after subcutaneous injection.^63^ By intravenous injection, the half-life of GM-CSF in mice is 6–8.6 h.^32^ In our study, we found that an albumin-fusion strategy significantly increased serum levels at 24 h, and our albumin-fusion strategy requires FcRn to target GM-CSF to LNs. The administration of albGM-CSF still increased the DC population in the lungs of FcRn-KO mice, indicating that an alternative mechanism may play an important role in the metabolism of albumin in the lungs. This effect may be related to the increase in molecular weight, which is above the threshold of kidney clearance.^64,65^ Further studies will be needed to elucidate the underlying mechanism(s) and pharmacodynamics of albGM-CSF under different administration routes.

GM-CSF promotes bone marrow cell proliferation and the expansion of macrophages and granulocytes.^66^ GM-CSF can enhance intracellular bacterial killing in vitro^46,47^ and is an important mediator for controlling intracellular *Mtb* infection via iNK T cells or keratinocyte growth factor.^46,47^ GM-CSF-deficient mice infected with *Mtb* had accelerated mortality and more extensive pulmonary necrosis than control *Mtb*-infected mice, and these findings were related to impaired Th1 responses in the former mice.^67^ During chronic TB infection in mice, GM-CSF expression is decreased relative to that during acute infection, and intratracheal administration of adenovirus encoding GM-CSF enhances *Mtb* control in acute and chronic infection.^30^ It has been shown that GM-CSF-producing T cells play an important role in controlling chronic TB.^25^ The utility of GM-CSF in controlling chronic TB has been shown in animal models,^29^ although limited efficacy was observed in a clinical trial,^31^ perhaps due to the suboptimal route and frequency of administration. In the current study, we used an albumin-fusion strategy to improve the bioavailability and targeting of GM-CSF to the lungs and dLNs of mice. During pulmonary TB, there is significantly increased transcytosis, supporting the potential role of this approach for targeting protein or drugs to the lungs.

The ability of GM-CSF to differentiate bone marrow cells into DCs in vitro is well characterized,^9,11^ as is its role in enhancing antigen presentation to T cells, thus supporting its potential utility as an adjuvant for TB vaccination.^16,20,68,69^ Its dual functions in both innate and adaptive immunity make GM-CSF a unique candidate in treating TB. Macrophages and DCs express the GM-CSF receptor, whereas T cells do not.^53^ We have shown that both GM-CSF and albGM-CSF can enhance bacterial control during macrophage infection ex vivo (Fig. 2d). This effect can directly decrease the bacterial burden in the lungs. Furthermore, it has been shown that GM-CSF can enhance LPS-induced IL-1β secretion from DCs and macrophages by activating NF-κB signaling.^58,59^ IL-1β is a key cytokine contributing to the control of *Mtb* growth during chronic infection.^70,71^ Here, we showed that IL-1β levels were significantly higher when *Mtb*-infected DCs or macrophages were treated with GM-CSF or albGM-CSF ex vivo than when they received no treatment. In a mouse model of chronic TB, albGM-CSF-treated mice displayed significantly higher levels of IL-1β in the lungs than untreated mice or mice treated with GM-CSF. In addition to IL-1β, it has been shown that GM-CSF can enhance tumor necrosis factor α and IL-6 secretion from LPS-stimulated DCs.^58^ Another important cytokine, IL-17, is associated with GM-CSF.^72,73^ IL-17-secreting CD4^+^ T cells are important for protection against TB,^73,74^ but Th17 responses are not essential for the control of disease when Th1 responses are intact.^75^ The role of IL-17 during chronic TB infection is uncertain^75^ and may relate to outcomes of chronic infections, such as granuloma formation.^76,77^ It is possible that there are other cytokines or mechanisms that contribute to the bactericidal effects of GM-CSF. Further in vivo studies to block the effects of IL-1β or other mediators, e.g., using neutralizing antibodies, will be required to determine the precise mechanism(s) by which GM-CSF exerts its antimycobacterial effects. Finally, more work is required in preclinical models to evaluate the potential utility of albGM-CSF as an adjunctive therapy in combination with the standard first-line regimen with the goals of shortening the duration of treatment for drug-susceptible and drug-resistant TB and improving TB-induced lung pathology.

## Conclusions

In the current study, we used an albumin-fusion strategy to enhance the delivery of GM-CSF to the lungs and dLNs of mice. We found that albGM-CSF acted as an in situ vaccine, increasing the number of DCs in the dLNs and lungs and yielding a tuberculocidal effect in the lungs. Further studies are needed to evaluate the potential utility of albGM-CSF as an adjunctive therapy in combination with the standard first-line regimen to shorten the duration of treatment for drug-susceptible and drug-resistant TB and to improve TB-induced lung pathology. In addition to its potential utility for chronic pulmonary infections, this albumin-fusion strategy may represent a promising approach for developing novel adjuvants in cancer prevention and therapeutic vaccines.

## Supplementary information

Supplemental Figure 1

Supplemental Figure 2

Supplemental Figure 3

Summary
