# Supplementary figures and images for: Albumin fusion with granulocyte-macrophage colony-stimulating factor acts as an immunotherapy against chronic tuberculosis
Source: Cell Mol Immunol. 2020 May 7;18(10):2393–401. doi: 10.1038/s41423-020-0439-2 (PMC8484439; doi:10.1038/s41423-020-0439-2)

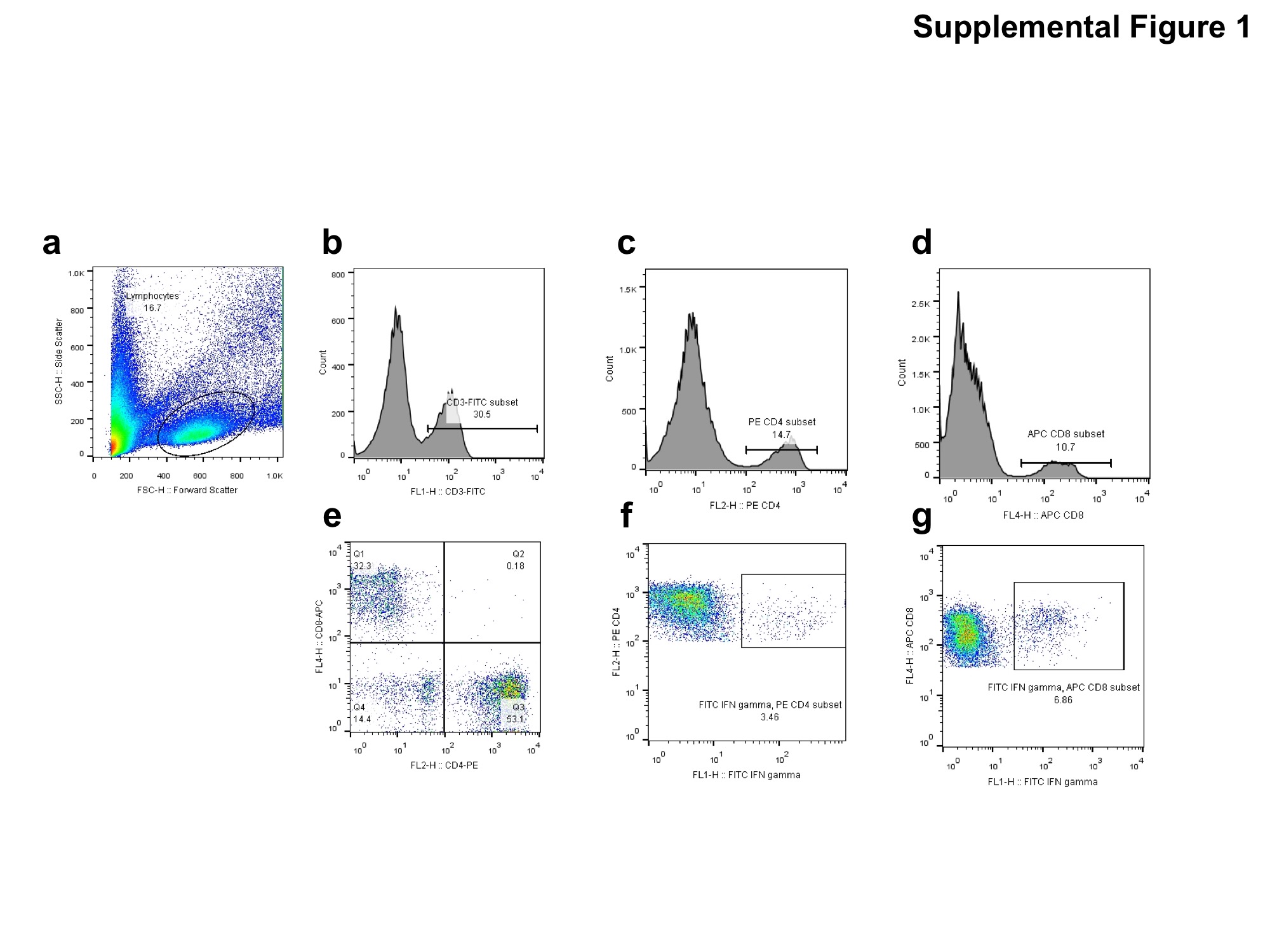

Supplement: Supplementary file 1 — Supplemental Figure 1 [file 41423_2020_439_MOESM1_ESM.jpg]

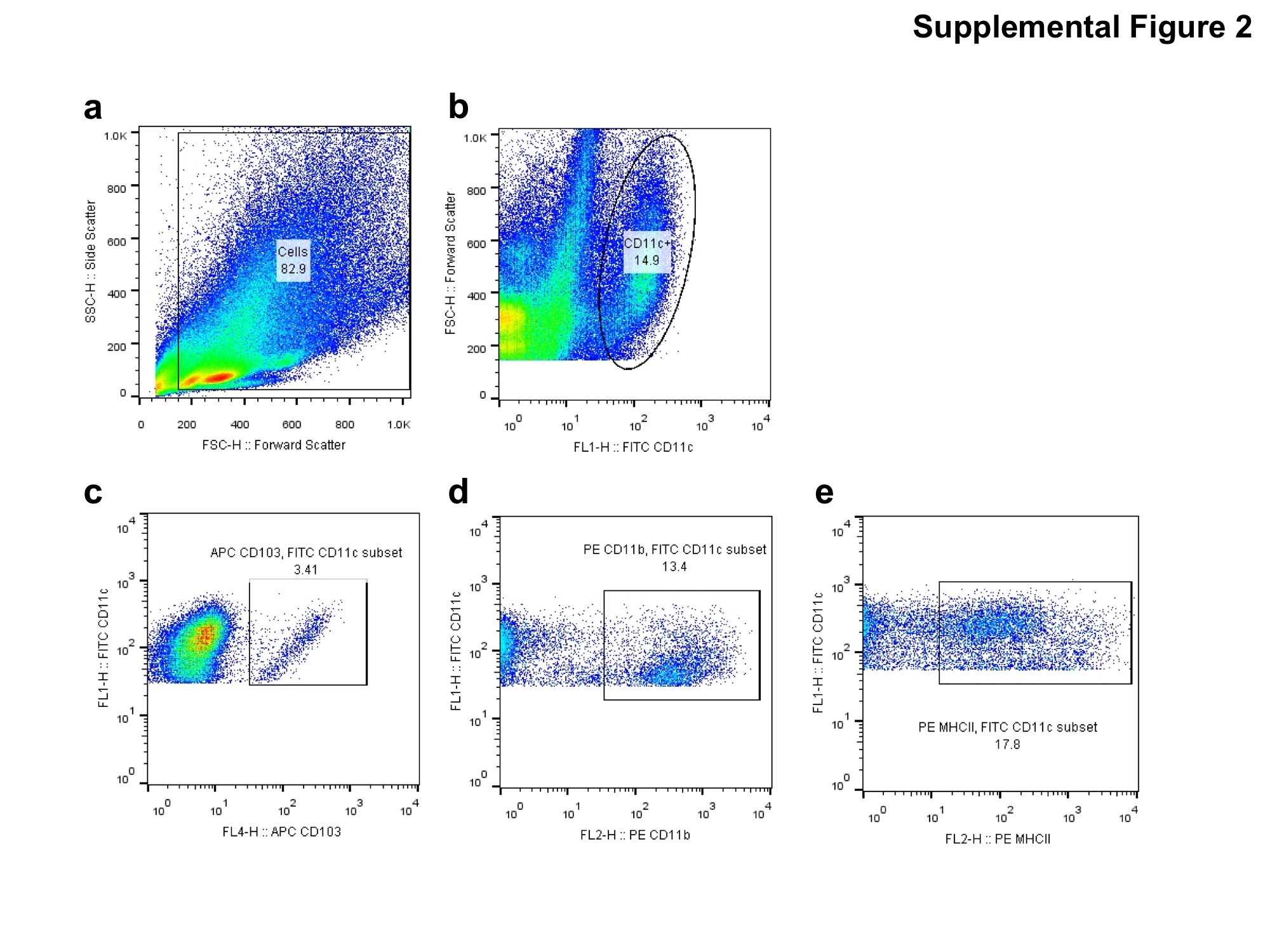

Supplement: Supplementary file 2 — Supplemental Figure 2 [file 41423_2020_439_MOESM2_ESM.jpg]

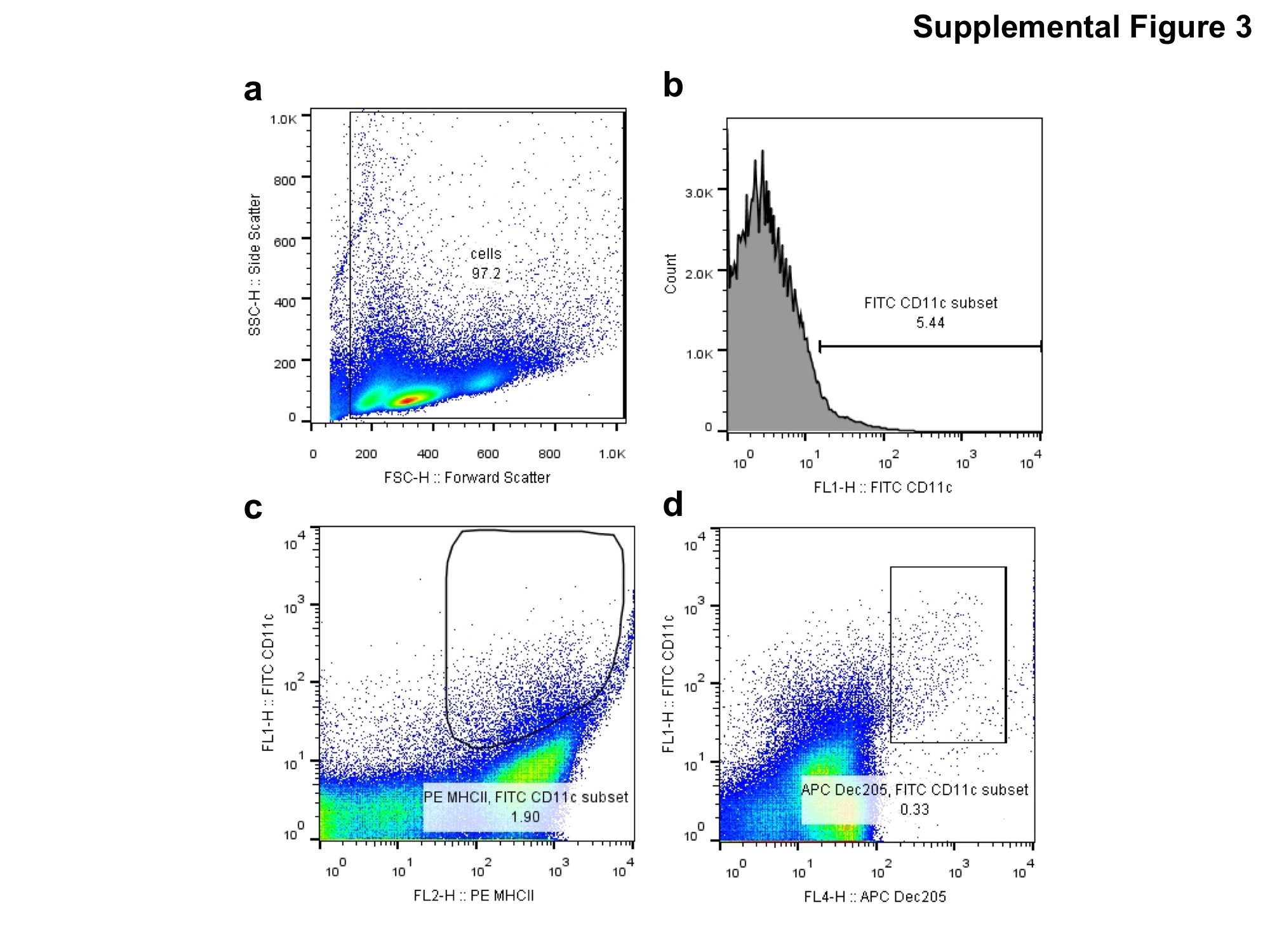

Supplement: Supplementary file 3 — Supplemental Figure 3 [file 41423_2020_439_MOESM3_ESM.jpg]

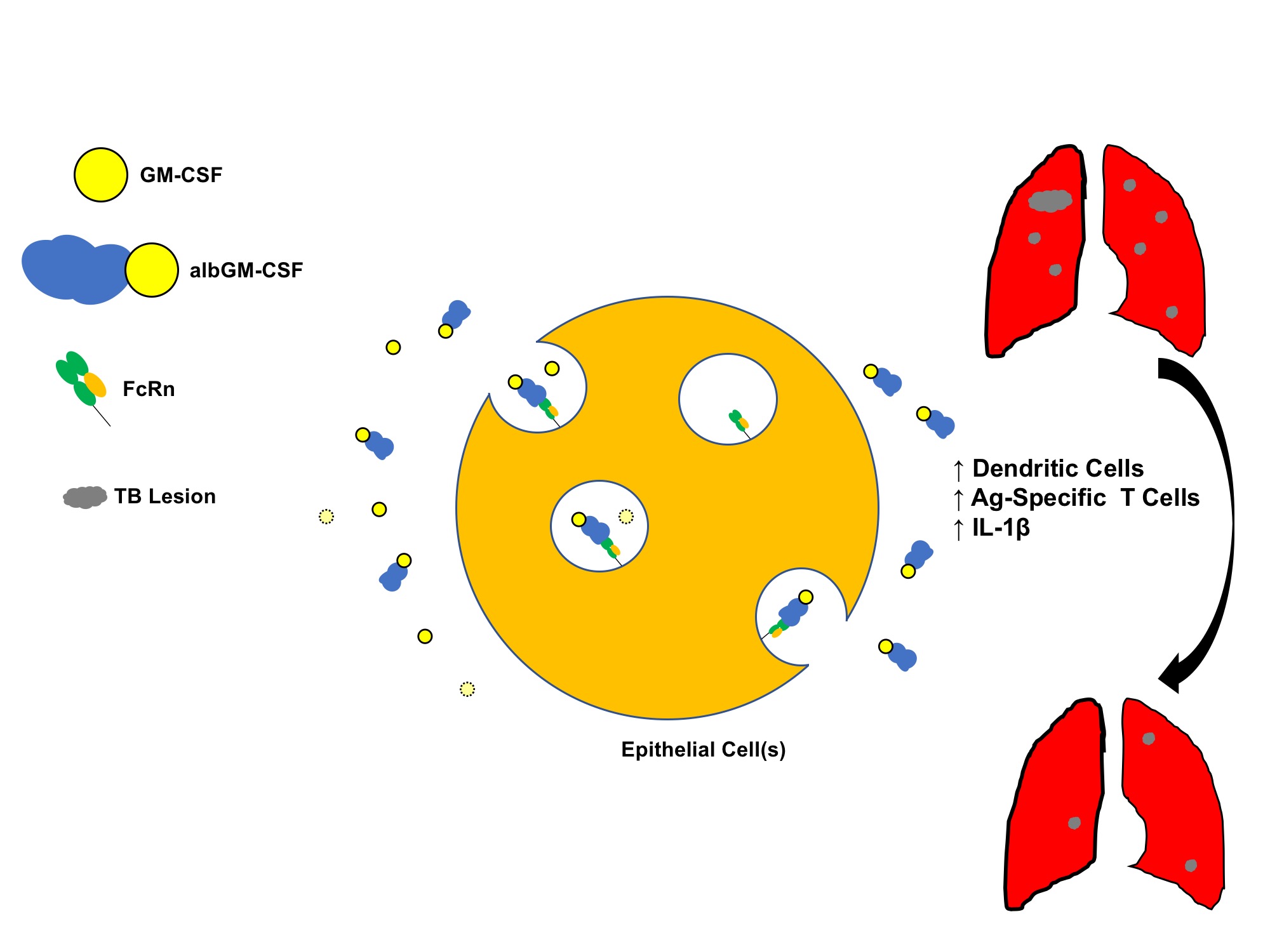

Supplement: Supplementary file 4 — Summary [file 41423_2020_439_MOESM4_ESM.jpg]
